# Identification of candidate genes for human retinal degeneration loci using differentially expressed genes from mouse photoreceptor dystrophy models

**Published:** 2008-09-05

**Authors:** Christina Demos, Mausumi Bandyopadhyay, Bärbel Rohrer

**Affiliations:** 1 Department of Physiology and Neuroscience, Medical University of South Carolina, Charleston, SC; 2 Department of Ophthalmology, Medical University of South Carolina, Charleston, SC

## Abstract

**Purpose:**

Retinal degeneration (RD) is a complex mechanism that appears to involve many biologic processes including oxidative stress, apoptosis, and cellular remodeling. Currently there are 51 mapped, but not identified, RD human disease loci.

**Methods:**

To assign possible disease genes to RD loci, we have used a comparative genomics procedure that incorporates microarray gene expression data of three independent mouse models for photoreceptor dystrophy (*rd1*, *rd2,* and constant light-damage in BALB/c mice), human ortholog data, and databases of known chromosomal locations involved in human RD. Immunohistochemistry and enzyme activity assays were used to further characterize a candidate gene product.

**Results:**

Our analysis yielded candidate genes for four mapped, but unsolved, human chromosomal locations and confirmed two previously identified monogenic disease loci for human RD, thus validating our approach. *PLA2G7* (phospholipase A2, group VII; PAF-AH, Lp-PLA2), a candidate for a dominant form macular dystrophy (Benign Concentric Annular Macular Dystrophy [BCMAD]), was selected for further study. The PLA2G7 enzyme is known to mediate breakdown of oxidatively damaged phospholipids, a contributor to oxidative stress in the retina. PLA2G7 protein was enriched in mouse photoreceptor inner and outer segments. In the *rd1*, *rd2*, and BALB/c mice exposed to constant light, retinal tissue activity levels, but not plasma levels, were significantly reduced at the onset of photoreceptor cell death.

**Conclusions:**

We have shown that this comparative genomics approach verified existing RD genes as well as identified novel RD candidate genes. The results on the characterization of the PLA2G7 protein, one of the novel RD genes, suggests that retinal tissue PLA2G7 levels may constitute an important risk factor for BCMAD. In summary, this reverse mapping approach, using accepted mouse models of human disease and known human RD loci, may prove useful in identifying possible novel disease candidates for RD and may be applicable to other human diseases.

## Introduction

The identification of genes and loci causing inherited retinal diseases such as retinitis pigmentosa (RP), macular degeneration (MD), and Usher (USH) syndrome are crucial for disease management [1]. Inherited retinal degeneration (RD) is the major cause of blindness in the developed world. Retnet lists 28 different categories, and two complex forms of retinal disease, including 191 loci that have been mapped; the disease gene has been identified for 140 of these loci. While great strides have been made to identify genes and mutations causing these diseases, progress has been hampered by their enormous complexity, due to genetic, allelic, phenotypic, and clinical heterogeneity of patient populations [2]. For example, autosomal dominant RP has been associated with mutations in 16 genes and another locus with the gene yet to be determined; autosomal dominant macular degeneration has been associated with mutations in 14 genes and eight other loci; and autosomal recessive RP has been associated with mutations in 21 genes with five other loci. Thus, it is clear that there are a good number of existing RD loci for which the mutant genes are yet to be determined and, in addition, yet to be discovered new loci for various monogenic and complex RDs such as age-related macular degeneration (AMD) and diabetic retinopathy.

We propose a technique to identify possible gene candidates for these human disease loci: gene expression analysis in mouse models of photoreceptor dystrophy. These analyses could identify genes that are misregulated during photoreceptor degeneration, correlating the human orthologs with chromosomal locations associated with inherited human retinal degeneration. Here we have followed this approach by using gene expression analysis from three unrelated mouse models of photoreceptor dystrophy. Two models match the human condition, as the same gene functions are affected (the *rd1* and the *rd2* mouse [3,4]) as well as a popular oxidative stress model thought to be relevant for diseases such as AMD (light-damage (LD) in albino mice [5]). The results are based on the premise that information collected about the orthologs of genes between species that exhibit the same trait or disease may be useful. Orthologs, by definition, evolved from the same gene, and usually share the same function. Correlating this information may provide evidence to determine a regulatory pattern of orthologs associated with congruent traits [6]. Genes that matched to the human RD loci and that were commonly up- or downregulated in all three models of degeneration are thought to be good candidates, especially if a literature search suggests that the known biologic information might be relevant to RD. Finally, as human retinal degenerations are usually caused by missense or nonsense mutations resulting in altered gene expression, the approach using expression differences to identify candidates is acceptable.

One of the genes, *PLA2G7* (PAF-AH, Lp-PLA2), a candidate for a dominant form of macular dystrophy, benign concentric annular macular dystrophy (BCMAD), was selected for further study. The main function of platelet-activating factor (PAF) acetylhydrolase is to convert PAF into the biologically inactive lyso-PAF [7]. However, PLA2G7 also hydrolyzes oxidized phospholipids. Oxidized phospholipids are known to initiate cell death, triggering the intrinsic apoptotic caspase cascade [8]. Oxidative stress is activated in the photoreceptors of the three models of retinal degeneration studied herein [9] and is a contributing factor in AMD [10]. In addition, products generated by PLA2G7, lysophosphatidylcholine and oxidized nonesterified fatty acids, are thought to contribute to inflammation in atherosclerosis, coronary artery disease, and stroke [11]. Plasma PLA2G7 activity levels can be used in these diseases as a biomarker, while also functioning as an independent risk predictor for cardiovascular disease [12]. Finally, inflammation has been proposed as a possible driving force of AMD pathology [13-18].

The reverse-mapping approach identified possible novel disease candidates for RD, which are discussed in the context of their known gene function and possible involvement in disease pathology. Of the identified candidate genes, two of them were previously confirmed to be the disease genes in loci associated with photoreceptor degeneration, supporting the validity of our approach while four additional genes were novel candidates for three mapped RD chromosomal loci. One of the candidate gene products, Pla2g7, was localized to the mouse photoreceptor inner and outer segments, and retinal tissue activity levels were significantly reduced before photoreceptor cell death. Hence, this tactic has resulted in the identification of novel candidates for three RD loci and demonstrated this as a feasible approach to identifying gene candidates for other human diseases as well.

## Methods

### Animals

C57BL/6 *rd1* [19] and *rd2* [4] mice were gifts from Drs. Debora Farber and Gabriel Travis (both at University of California, Los Angeles, CA). Both strains were maintained as homozygotes. C57BL/6 and BALB/c mice were generated from breeding pairs obtained from Harlan Laboratories (Indianapolis, IN). Animals were housed in the Medical University of South Carolina (MUSC) Animal Care Facility under a 12 h:12 h light–dark cycle with access to food and water ad libitum. The ambient light intensity at the eye level of the animals was 85±18 lux. Light damage was produced by exposing the BALB/c animals to constant fluorescent light for 24 or 48 h at an illuminance of approximately 1500 lux. This intensity reduces the number of photoreceptors to 50% within 10 days in 3-month-old (young adult) albino mice [5]. All experiments were performed in accordance with the ARVO Statement for the Use of Animals in Ophthalmic and Vision Research and were approved by the University Animal Care and Use Committee.

### Microarray analyses

#### Samples

Affymetrix oligonucleotide (MGU74AV2) arrays (Affymetrix Inc, Santa Clara, CA), containing 12489 genes and ESTs, were used for this analysis as described previously [20]. The Affymetrix CEL files, containing the raw intensity values, were used for expression data analysis. To determine genes that could be potential candidates for retina-specific chromosomal locations, we compared gene expression data from the three unrelated mouse models of photoreceptor dystrophy. To analyze genes involved in neurodegeneration, we argued that genes altered early in the progression would be involved in initiating degeneration. For the *rd1* mouse, we collected retinas from days P6 and P10, which represent early time points during which cGMP continues to rise [3] and apoptosis is initiated [21]; for the *rd2* mouse, we collected retinas from P14 and P21, representing early time points during the first phase of apoptosis [21]; and finally for the light-damaged paradigm, we collected retinas 24 and 48 h after the onset of constant light at 1500 lux, a point at which a few TUNEL-positive photoreceptors can be observed, but no cell loss can yet be documented [9].

#### RNA isolation

All chemicals used in this study were at least molecular biology grade material and purchased from Fisher Scientific (Pittsburgh, PA), unless otherwise noted. Animals (see Samples for ages of animals) were sacrificed by decapitation and retinas isolated and stored in RNA-later (Ambion, Austin, TX) at −20 °C. Retinas from four animals per genotype per time point were pooled, and each data point was obtained in duplicate. Pooling is recommended as the method of choice to reduce the number of arrays needed to generate reliable data [20,22]. Total RNA was isolated using Trizol (Ambion), followed by a clean-up using RNAeasy minicolumns (Qiagen, Valencia, CA). The quality of the RNA was examined by gel electrophoresis, and spectrophotometry [20].

#### Microarray procedures

Sample preparation and hybridization was performed as described in the Affymetrix Expression Analysis Technical Manual and published previously [20]. In short, double-stranded cDNA was generated (SuperScript™ II Reverse Transcriptase; Invitrogen, Carlsbad, CA) using 5 μg total RNA as starting material, and purified using phase-lock gel columns (Eppendorf, Westbury, NY) followed by ethanol precipitation. The purified cDNA served as a template for the generation of biotinylated cRNA, using the BioArray™ HighYield™ RNA transcript labeling kit (Enzo Diagnostics, New York, NY). Labeled probes were purified using the RNEasy mini kit (Qiagen, Valencia, CA), fragmented by metal-induced hydrolysis at 94 °C for 35 min (100 mM potassium acetate, 30 mM magnesium acetate, and 40 mM tris-acetate) and stored at -80 ºC. The length of the cRNA and fragmentation was confirmed by agarose gel electrophoresis. Hybridization with equal amounts of labeled cRNA (15 µg/array) and readout was performed by the DNA Microarray Core Facility at MUSC, using the Affymetrix Fluidics Station.

### Data analysis

#### Normalization and filtering

Genechips were scanned using the Affymetrix scanner (Microarray Suite 5.0 software) to obtain probe level data. Outputs were scaled to the same target intensity. The raw Affymetrix data (absolute expression level and perfect match (PM)-values) was used for normalization. Each of the three model sets were normalized using quantile normalization on the probe and probe set level. This procedure was done using Dchip software [23]. Gene filtering was performed individually on the three retinal degeneration sets. Normalized data was filtered on significant p-values (≤0.05) in fold change and difference of the means between experimental and age-matched control samples (value of ≥100). With an estimated median expression level of 90 this automatically excludes low-expressing genes. Venn analysis was used to identify genes that localized to RD loci.

#### Analysis of retinal degeneration chromosomal loci

For us to be able to match the differentially expressed mouse genes to known chromosomal locations involved in retinal degeneration, we needed human orthologs to these mouse genes. The Affymetrix NetAffx Analysis Center was used to obtain the human orthologs, as well as accession numbers and chromosome locations for all genes. The list of human ortholog locations was correlated with the 191 human retina-specific locations currently listed in RetNet, to determine which locations were unknown and unsolved.

#### Gene ranking and probability

To determine the probability of one of the genes in our analysis falling into one of the retina-related loci, we implemented an algorithm using the gene lengths, locus lengths, and chromosome lengths. Probability was determined by calculating the ratio of gene length to locus length over the ratio of the gene length to chromosome length. Genes were ranked based on a combined score of probability: 5 (0%–4.9%), 4 (5%–9.9%), 3 (10%–14.9%), 2 (15%–19.9%), 1 (20%-above). This score was multiplied by the number of models in which the genes were differentially expressed (3, 2, or 1), resulting in a maximum score of 15.

#### Gene ontology analysis

Gene Ontology (GO) analysis on the identified genes was done using GoStat by Tim Beissbarth. GO p-values were computed, and the GO terms with significant p-values identified to compile the final list of overrepresented GO terms. All ontologies (Molecular Function, Biologic Process, and Cellular Component) were analyzed as a group.

### Pla2g7 analysis

#### Immunohistochemistry

For immunohistochemical analysis, eyes were fixed in 4% paraformaldehyde, rinsed, cryoprotected in 30% sucrose overnight, frozen in TissueTek O.C.T. (Fisher Scientific) and cut into 14 μm cryostat sections. Immunohistochemistry was performed as described previously [24] using an anti-PAF-AH antibody (Lis-1; Abcam, Cambridge, MA) at 1:100. For visualization, a fluorescent-labeled secondary antibody (Alexa 488; Invitrogen, Carlsbad, CA) was used. Each staining was performed on slides from at least three animals per condition. Sections were examined by fluorescence microscopy (Zeiss) and images were false-colored using Adobe^®^ Photoshop (Adobe Systems, San Jose, CA).

#### Activity assay

PLA2G7 is known to catalyze the hydrolysis of the substrate platelet-activating factor (PAF) into the biologically inactive lyso-PAF. The assay (Cayman Chemical, Ann Arbor, MI) uses 2-thio PAF as a substrate for PAF-AH. Hydrolysis produces free thiols, reacting it with an excess of 5,5‘-dithio-bis-2-nitrobenzoic acid (DTNB); which is measured spectrometrically. Neither the substrate nor the lyso-PAF react with DTNB. As a negative control the enzyme source (plasma or retina) is heat-inactivated for 15 min and used with the substrates; human PLA2G7 provided in the kit was taken as positive control for all the measurements. The commercial kit was used according to the manufacturer’s recommendations.

For tissue levels, retinas were dissected out from eyes of *rd1* (P10), *rd2* (P21), and 48 h light-damaged BALB/c mice and corresponding control animals. Retinas were homogenized in 100 μl of cold Tris-Cl buffer (0.1 M, pH 7.2) and centrifuged at 10,000x g for 15 min at 4 °C. Supernatants were collected and total protein content in each sample assayed by the Bradford method. To determine plasma levels, blood was collected from the submandibular vein in isoflurane anesthetized mice. The vein was punctured with a 22 gauge needle, which initiates blood flow and sample collected with a pipette using citrate as an anticoagulant (0.38% final concentration). Plasma samples were collected after centrifugation (800x g for 10 min at 4 °C).

The assay-mixtures each contained 10 μl of sample, to which 5 μl of assay buffer was added to each well of a 96 well flat-bottom plate. Reaction in each well was initiated by adding 200 μl of substrate solution (2-thio PAF). Following incubation at room temperature (30 min for retina, 1 min for plasma), 10 μl of DTNB was added to each well. Color development was measured in a spectrophotometer (Softmax; Molecular Devices, Sunnyvale, CA) at 405 nm, 1 min after the addition of DTNB. Specific activity of PLA2G7 was calculated from the absorbance values (extinction coefficient for DTNB at 405 nm, 12.8/mM/cm). Data are expressed as mean±SE of at least three independent Pla2g7 activity measurements in units of specific activity for tissue [μmol/minute/mg of protein] or plasma [μmol/minute/ml of plasma].

## Results

### Identification of candidate genes

The RetNet database currently lists 191 retina-specific human loci: 140 of the human disease loci are mapped and the disease gene identified, leaving 51 of the human loci uncharacterized (see RetNet). By correlating the nucleotide position data for each of these unknown locations with those of the 12489 genes and ESTs present on the MGU74Av2 array and their orthologs, we have the potential to identify candidate genes for 37 of these unsolved disease loci (approximately 73%).

Mapped but unidentified chromosomal disease loci are typically large, some spanning many cM, and harbor upwards of hundreds of genes. For example, the 37 RD loci for which genes matched in the MGU74Av2 array range in size from 1.8 to 49.2 Mbp (median size: 16.71 Mbp). The average number of genes contained within a location of 16.71 Mbp is 393.18, based on an average gene density of 40–45 kb [25]. Identifying potential candidates requires additional search criteria. Underlying an identification of a mapped locus are genetic differences influencing the susceptibility to a trait or disease. Thus, here we argued that these presumed genetic differences should be reflected in the difference in retinal gene expression of mice with RD.

To analyze differences in gene expression related to photoreceptor degeneration, we selected three unrelated mouse models of photoreceptor dystrophy: the *rd1* mouse (calcium overload) [26]; the *rd2* mouse (structural defect due to a mutation in the disc rim protein peripherin); and constant light-damage (LD; oxidative stress) [3,5,27]. The *rd1* mouse is considered a model for RP, whereas the *rd2* mouse and the LD model are used as models for both RP and macular degeneration. For each mouse model, we determined changes in gene expression between the experimental animals and their age-matched controls at two consecutive time points early in the progression of degeneration. For a given gene to be considered as a possible candidate or a retina-specific location, it had to be significantly up- or downregulated (p<0.05) with a predefined mean difference in expression level (≥100) in at least one of the three models. Of the 902 genes that met these criteria, 20 genes were found to have human orthologs that were localized to human retinal degeneration (Table 1). Experimental data regarding gene expression levels and fold differences in gene expression (Appendix 1) are provided in the supplemental material section. These 20 genes were ranked on two criteria: (a) based on their probability of falling within a human disease locus by chance; (b) multiplied by the number of models in which the genes were differentially expressed. Due to the significant difference in locus size for the different diseases (i.e., the 20 loci range in size from 1.7 to 60 Mbp), the probability ranged from 1.9% to 46.9% (Table 2, column 5), with the median probability of 6.6%.

**Table 1:** Genes corresponding to human disease loci identified by being either commonly up- or down-regulated in three, independent mouse models of photoreceptor dystrophy.

**Affymetrix** **gene ID**	**Gene name**	**Gene** **symbol**	**Mouse** **transcript** **ID**	**Human ortholog NM**	**Ortholog** **chromosomal** **location**	**Retinal degeneration locus**
160901_at	FBJ osteosarcoma oncogene	*Fos*	NM_010234	NM_001040059	14q24.3	**LCA3**
97540_f_at	histocompatibility 2, D region locus 1	*H2-D1*	NM_001025208	NM_005516	6p21.3	TULP1/RP14
101923_at	phospholipase A2 group VII (platelet-activating factor acetylhydrolase, plasma)	***Pla2g7***	NM_013737	NM_005084	6p21.2-p12	RDS/RP7; GUCA1A, GUCA1B; **BCMAD**
98549_at	vitronectin	*Vtn*	NM_011707	NM_000638	17q11	UNC119/HRG4
98579_at	early growth response 1	***Egr1***	NM_007913	NM_001964	5q31.1	**BSMD**, PDE6A
92223_at	complement component 1, q subcomponent, C chain	*C1qc*	NM_007574	NM_172369	1p36.11	NRL/RP27
96020_at	complement component 1, q subcomponent, beta polypeptide	***C1qb***	NM_009777	NM_000491	1p36.3-p34.1	**LCA9, RP32**
103033_at	complement component 4 (within H-2S)	*C4*	NM_009780	NM_000592	6p21.3	TULP1/RP14
98472_at	histocompatibility 2, T region locus 23	*H2-T23*	NM_010398	NM_005252	6p21.3	TULP1/RP14
94701_at	phosphodiesterase 6B, cGMP, rod receptor, beta polypeptide	*Pde6b*	NM_008806	NM_000283	4p16.3	PDE6B/CSNB3, MCDR2
102612_at	neural retina leucine zipper gene	*Nrl*	NM_015810	NM_006177	14q11.1-q11.2	NRL/RP27
160894_at	CCAAT/enhancer binding protein (C/EBP), delta	***Cebpd***	NM_007679	NM_005195	8p11.2-p11.1	**CORD9**
94854_g_at	guanine nucleotide binding protein, beta 1	***Gnb1***	NM_008142	NM_002074	1p36.3-p34.1	**LCA9, RP32, RD4**
93120_f_at	histocompatibility 2, K region	*H2-K*	NM_001001892	NM_002127	6p21.3	TULP1, RP14
98562_at	complement component 1, q subcomponent, alpha polypeptide	***C1qa***	NM_007572	NM_015991	1p36.3-p34.1	**LCA9, RP32**
95974_at	guanylate nucleotide binding protein 1	*Gbp1*	NM_008142	NM_002074	1p36.3-p34.1	ABCA4
103202_at	guanylate nucleotide binding protein 3	*Gbp3*	NM_018734	NM_133263	1p22.2	ABCA4
103634_at	interferon dependent positive acting transcription factor 3 gamma	*Isgf3g*	NM_008394	NM_006084	14q11.2	NRL/RP27
104669_at	interferon regulatory factor 7	*Irf7*	NM_016850	NM_004030	11p15.5	TEAD1/AA/TCF13/ TEF1
99608_at	peroxiredoxin 2	*Prdx2*	NM_011563	NM_005809	19p13.2	R9AP

The highlighted gene symbols (column 3) represent the mapped, but unsolved loci (column 7), the remaining genes localize to loci that have already been solved. The genes are documented with respect to the mouse gene name, symbol and mouse transcript ID (columns 2-4) and their human ortholog (column 5). Column 6 provides information about the chromosomal location of the human ortholog; column 7 lists the name(s) of the loci. Please note that in some entries in the locus column, there are multiple names given, meaning that more than one trait resides in that chromosomal location; however these may or may not be related. Column 1, the Affymetrix Gene ID; and column 3, the gene symbol; represent the common denominators for all Tables in the manuscript (Table 1 and Table 2) and Appendix 1 and Appendix 2.

**Table 2:** Characterization of genes identified as candidates for human disease loci.

**Affymetrix gene ID**	**Gene symbol**	**Mis-regulation**	**Animal models**	**Probability**	**Score**	ONL: sage, qRT-PCR*	**injury**	**Bright LD #**	**Retina network**
160901_at	***Fos***	up	*rd1/rd2*/LD	0.044	15	x*	+		1
97540_f_at	*H2-D1*	up	*rd1/rd2*/LD	0.046	15	x			3
101923_at	***Pla2g7***	down	*rd1/rd2*/LD	0.046	15	x			2
98549_at	*Vtn*	down	*rd2*/LD	0.019	10	x			2
98579_at	***Egr1***	up	*rd1/rd2*	0.132	6	x*	+		1
92223_at	*C1qc*	up	*rd1/rd2*	0.143	6	x	+	+	3
96020_at	***C1qb***	up	*rd1/rd2*	0.116	6	_*	+		3
103033_at	*C4*	up	*rd2*	0.046	5	?	+	+	3
98472_at	*H2-T23*	up	*rd2*	0.046	5	?		+	3
94701_at	*Pde6b*	down	*rd1/rd2*	0.179	4	x			2
102612_at	*Nrl*	down	*rd2*	0.066	4	x			2
160894_at	***Cebpd***	up	*rd1/rd2*/LD	0.238	3	_	+	+	1
94854_g_at	***Gnb1***	down	*rd2*/LD	0.135	2	x			2
93120_f_at	*H2-K*	up	*rd2*/LD	0.046	2	x		+	3
98562_at	***C1qa***	down	*rd2*	0.135	1	_		+	3
95974_at	*Gbp1*	down	*rd2*	0.0338	1	?		+	na
103202_at	*Gbp3*	down	*rd2*	0.0338	1	x			na
103634_at	*Isgf3g*	up	*rd2*	0.066	1	x		+	3
104669_at	*Irf7*	down	*rd2*	0.0611	1	_		+	3
99608_at	*Prdx2*	down	*rd2*	0.4691	1	?			na

Genes are identified by Affymetrix Gene ID (column 1) and gene symbol (column 2) for easy comparison with Table 1. Column 3 identifies the type of misregulation (up- or down-regulated) and column 4 documents in which animal models the misregulation occurs. The probability of each gene to fall within the respective locus is listed in column 5; this probability multiplied by the number of models in which the genes are differentially expressed (3, 2, or 1) produced a gene ranking score (maximum column 6). The remaining columns document whether the respective gene is present in photoreceptors based on the retina SAGE library (column 7: x, present; -, absent; ?, no data available; *confirmed by qRT-PCR [9]), whether the gene is misregulated in retina injury models (column 8; identified by +) or after bright light exposure (column 9; identified by +), or which genes were found to cluster together (eye database at Genenetwork; column 10; 3 clusters, 1-3 were identified, as well as three unclustered genes).

### Gene ontology analysis of identified genes

To gain biologic understanding from the identified genes found in unsolved chromosomal locations (Table 1), we analyzed their functional annotations. GO identifications (GO IDs) and GO terms were retrieved for all significant ontologies (Biologic Process, Molecular Function, and Cellular Compartment). The GO terms associated with the 20 identified genes were compared to those of the reference group (all genes present on the array minus those listed in Table 1), determining significantly over-represented terms and obtaining important GO terms that describe these differentially regulated genes. The significantly overrepresented GO terms that were retrieved for the upregulated genes included the terms “defense response,” “immune response,” and “complement activation,” whereas in the downregulated genes, the terms identified the keywords “positive gene regulation of rhodopsin,” “retinal rod cell development,” and “thioredoxin peroxidase activity” (Appendix 2).

### Pla2g7 in retinal degeneration

**Figure 1:**
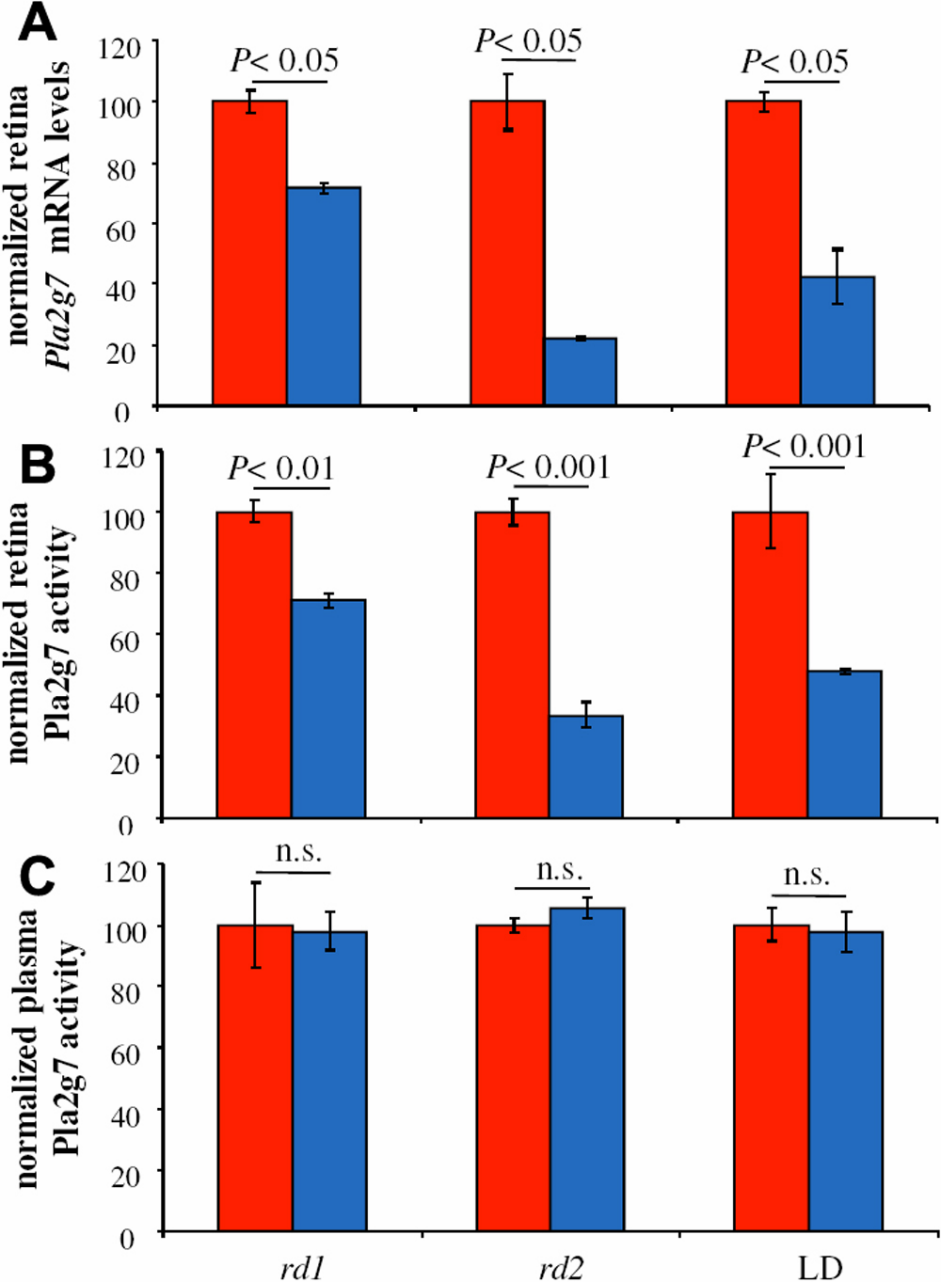
Pla2g7 mRNA and activity levels, analyzing levels from P10 *rd1*, P21 *rd2,* and 48 h light-exposed BALB/c animals and their respective age-matched controls. **A**: *Pla2g7* mRNA levels were plotted from Appendix 1. Retina *Pla2g7* mRNA levels are significantly reduced in all three genotypes when compared to controls. Data are expressed as mean±SD of the two arrays analyzed per genotype. **B**: Tissue retina Pla2g7 levels as measured in a calorimetric assay using 2-thio platelet activating factor (PAF) as substrate, revealed that activity levels in retinas from the three genotypes correlated well with the respective reduced amount of mRNA found in the tissue. Data are expressed as mean±SEM of at least three, independent samples in unit of activity (μmol/min/mg of protein). **C**: Plasma Pla2g7 levels measured in mandibular blood samples revealed that the two genetic mutations (*rd1* and *rd2*) or the environmental stress (constant light) did not influence systemic, plasma-derived Pla2g7 activity. Data are expressed as mean±SEM of at least three independent samples in unit of activity (μmol/min/mL of plasma). In the graph, red indicates control and blue indicates experimental. The following abbreviations were used: light-damage (LD) and not significant (n.s.)

*Pla2g7* (PAF-AH, Lp-PLA2), a possible candidate for a dominant form of macular dystrophy (BCMAD), was selected for further study. *Pla2g7* mRNA levels are significantly down-regulated in P10 *rd1* [fold difference (lower bound; upper bound)] [-1.39 (-1.26; -1.54)], P21 *rd2* [-4.5 (-3.5; -5.55)] and 48 h of light-damage in the BALB/c mouse retina [-2.37 (-1.57; -4.7)], which is before significant cell loss [9] (see Figure 1A). Pla2g7 localization in ocular tissues and PAF-AH activity levels in plasma and retina tissue were investigated.

**Figure 2:**
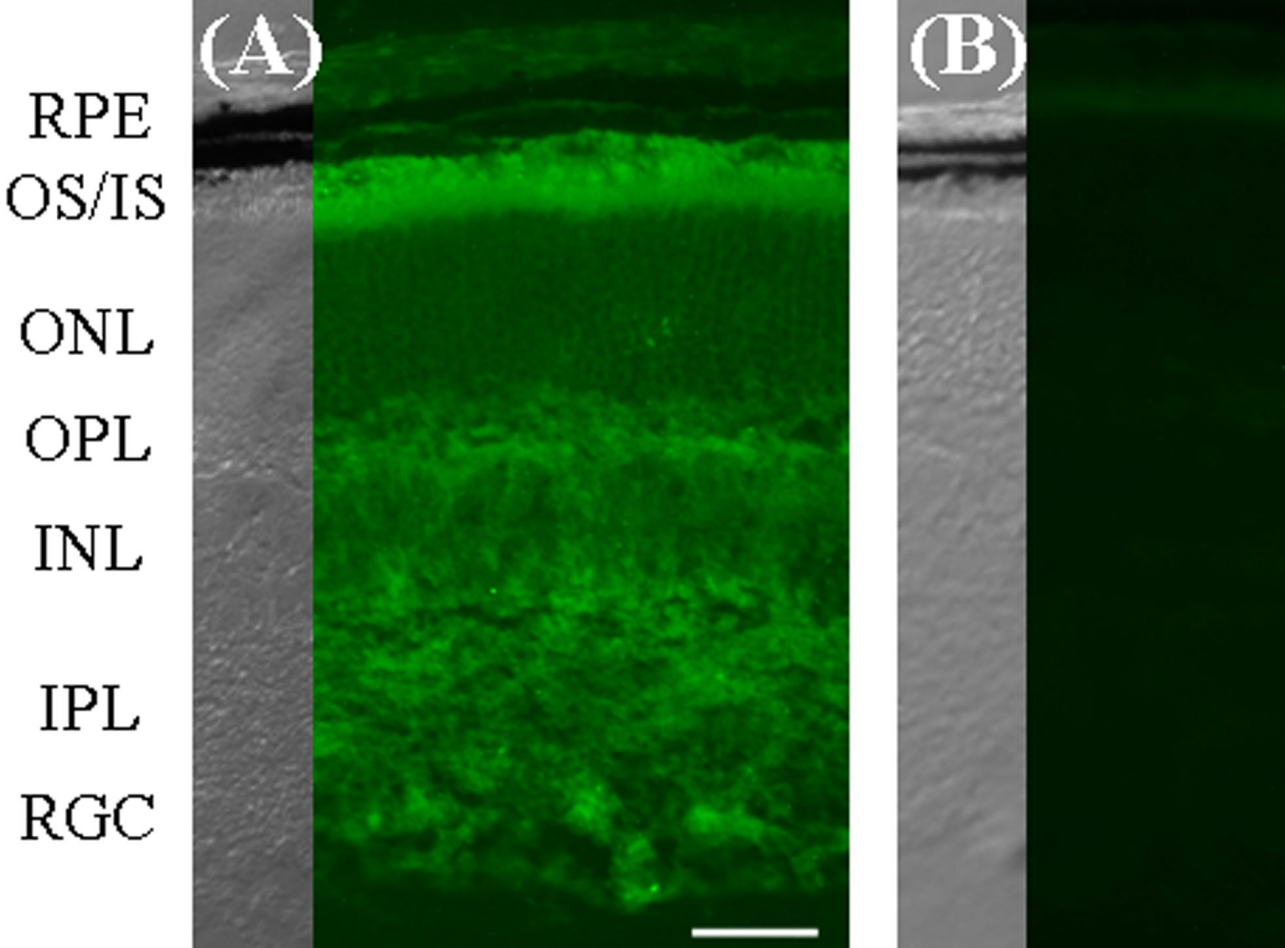
Pla2g7 localization. Immunohistochemistry was performed in juvenile C57BL/6 (P17) frozen sections (**A**), using no primary antibody conditions as the negative control (**B**). Pla2g7 was found to be localized throughout the retina. Relatively elevated levels were found in the photoreceptor inner and outer segments, whereas moderate staining was found in the two plexiform layers, as well as the inner nuclear layer (INL) and the retinal ganglion cell (RGC) layer. For each image, the corresponding DIC image is provided. The following abbreviations were used: retinal pigment epithelium (RPE), outer segments (OS), inner segments (IS), outer nuclear layer (ONL), outer plexiform layer (OPL), inner nuclear layer (INL), inner plexiform layer (IPL), and RGC: retinal ganglion cells (RGC). Scale bar in (**A**) represents 20 μm.

*Pla2g7* is a gene highly enriched in the mouse retina according to the Brain Gene Expression Map; in the retina, *Pla2g7* mRNA is present in the outer nuclear layer (ONL) that contains only the cell bodies with the nuclei of rods and cones (retina SAGE library) [28]. Immunohistochemistry revealed labeling in the photoreceptors (Figure 2), in particular the inner and outer segments, with additional labeling in the outer and inner plexiform layer, as well as staining of cells in the inner retina.

Pla2g7 activity was compared in soluble extracts of retina and in plasma (Figure 1B,C). Serum levels of Pla2g7 activity were not affected by RD triggered by either genetic (*rd1*, *rd2*) or environmental insults (LD; Figure 1C). Plasma levels in the P10, C57BL/6 wild-type mouse were higher than that obtained at P21, but not different from *rd1* at P10 nor *rd2* at P21 ([in μmol/min/ml plasma] P10: *wt*, 0.093±0.013 versus *rd1*, 0.091±0.0058; P21: *wt*, 0.0367±0.00009 versus *rd2*, 0.0387±0.0012). Likewise, no difference was identified in light-damaged BALB/c retina (cyclic light, 0.0935±0.005 versus LD, 0.0913±0.006). When compared to their respective age-matched controls, Pla2g7 levels were reduced by ~30% in the *rd1* retinas, by ~70% in the *rd2* retinas, and by ~50% in the light-damaged retinas (Table 3). Relative changes in retina *Pla2g7* mRNA levels were a good predictor of retina cytosolic Pla2g7 activity levels.

**Table 3:** Enzyme activity for Pla2g7 in retinal degeneration

**Genotype treatment**	**Control**	**Experimental**	**p-value**
*rd1*	0.2753±0.01	0.1953±0.006	<0.01
*rd2*	0.0805±0.0035	0.027±0.0.0032	<0.001
light damage	0.123±0.015	0.059±0.0012	<0.001

Quantification of specific activity of Pla2g7 [μmol/minute/mg of protein] in retina extracts collected from P10 *rd1*, P21 *rd2* mice, and 3-month old BALB/c mice after 24 h of light damage (column 2), together with their age-matched controls (column 3). Cytosolic levels of Pla2g7 were significantly reduced (column 4) in all three models of photoreceptor degeneration. Data is expressed as mean±SEM for 3-5 samples per condition.

## Discussion

### Comparative genomics analysis to identify novel disease genes

RD-causing mutations are found in genes whose proteins participate typically in one of four mechanisms: outer segment morphogenesis, cellular metabolism, function of the retinal pigment epithelium, and the photoreceptor signal transduction cascade [29]. However, other genes that are not typical photoreceptor-specific genes have been identified to have mutations in inherited RD, which include mutations in components of the alternative complement pathway (part of the body’s innate immune system) that have been shown to be associated with AMD [14,16-18]. Hence, we used a comparative genomics approach to aid in the identification of potentially novel disease genes.

Herein we have used gene expression analysis in three independent models of photoreceptor dystrophy, which showed key pathologies also seen in the human conditions, to identify novel candidates for gene loci known to be associated with inherited retinal diseases. While it would have been beneficial to obtain retina-specific arrays for our analysis, the U74Av2 arrays used had a present rate (i.e., genes that are expressed in the retina) of >50%, representing >6000 genes/ESTs. These 6,000 elements cover an estimated 50% of the 13k mammalian retinal transcriptome, as defined by Schulz and colleagues [30] or approximately 62% of the mouse retinal transcriptome identified by Blackshaw and coworkers [28]. Thus, our proposed “fishing expedition” still presents tremendous advantages when compared with a hypothesis-driven data analysis that investigates one gene at a time, but will likely miss roughly 50% of potential candidates.

To identify novel genes, we carefully filtered the genes that matched human RD loci to eliminate false positives, resulting in 20 potential gene candidates. These genes were ranked to focus on genes that have a low probability of falling within a region of interest by chance. To corroborate the potential for these 20 identified genes to be part of the molecular signature of photoreceptors and potentially prime candidate genes for human disease, the genes were further characterized based on their known retinal expression patterns. First, the list of genes was entered into the eye database at Genework to determine which genes would be correlated based on gene expression in the eye, within the BXD strains of mice along with the mouse diversity panel. Three subnetworks were identified (Table 2, column 10); a photoreceptor-specific network (*Pla2g7*, *Gnb1*, *Pde6β*, *Vtn*, and *Nrl*), transcription factors (*Fos*, *Egr1*, and *Cebpδ*), and one specific for immune-response (*H2-D2*, *C1qβ*, *C1qc*, *H2-K*, *C1qα*, *C4*, *H2-T23*, *Isgf3g,* and *Irf7*), as well as three unassociated genes (*Gbp1*, *Gbp3*, and *Prdx2*). Second, to determine whether these genes were expressed in the normal photoreceptors, we examined whether they were expressed in the outer nuclear layer (rods and cones) as assessed by Blackshaw and colleagues [28] using a mouse retina SAGE library or our own quantitative RT–PCR data on mouse ONL [9] (Table 2, column 7). All but one of the genes that were identified in at least two out of three models were found to be present in the photoreceptors, for a total of 13 out of 20. Three out of 20 genes were found to be absent, and no information was available for the remaining four out of 20 genes. Third, this set of 20 genes was compared with genes identified to be misregulated under unique retinal injury conditions such as diabetes [31], ischemia-reperfusion injury [32], retinal tears [33], elevation of intraocular pressure [34], laser-induced injury [35], photoreceptor degeneration induced by a photoreceptor-specific cadherin knockout [36], as well as a model of bright-light damage [36] (Table 2, columns 8 and 9). As expected, more extensive overlap was observed with bright-light-damage-induced genes, as one of our models was the constant, low-light-induced photoreceptor cell death model (*C1qα*, *C4*, *Gbp1*, *H2-K1*, *H2-T2B*, *Irf7*, *Isgf3g,* and *Nrl*); however, few genes were found to overlap with the general retinal injury models (diabetes: none; ischemia-reperfusion injury: none; retinal tears: *Egr1*, *Fos*, *C1qβ*, and *Cebpδ*; elevation of intraocular pressure: *Egr1*, *Cebpδ*; laser-induced injury: none) or with the photoreceptor cadherin knockout (*C4* and *Cebpδ*). Thus, it appears that the transcription factors *Fos*, *Egr1,* and *Cebpδ* are induced during general retinal injury, as is the complement system (*C4* and *C1qβ*). In summary, the final list of genes should have a high potential of detecting photoreceptor-specific disease genes.

### Candidate genes for retinal disease

Twenty genes passed our stringent selection criteria. Two out of the 20 genes confirmed monogenic loci associated with photoreceptor degeneration, which are typically named for the one gene carrying mutations responsible for disease (i.e., NRL and PDE6B), demonstrating that our method is able to identify previously characterized human retinal disease genes, and thus confirming the validity of our approach. Fourteen genes were identified that fell within the boundaries of the monogenic locus for which the responsible gene has already been identified, and are thus considered innocent bystanders: Locus (identified gene) TULP1 (*H2-D1*, *H2-K*, *C4*, *H2-T23*), RDS/RP7 (*Pla2g7*); GUCA1A (*Pla2g7*), GUCA1B (*Pla2g7*), NRL (*C1qc* and *Isgf3g*), UNC119 (*Vtn*), PDE6A (*Egr1*), MCDR2 (*Pde6β*), ABCA4 (*Gbp1* and *Gbp3*), R9AP (*Prdx2*), TEAD1 (*Irf7*). The eight remaining genes are potential candidates for mapped disease loci (Table 1). After subtracting those genes that were determined to be injury-related genes (*Fos*, *C1qβ*, *Cebpδ*, and *Egr1*), four potential genes remained. One of those genes was differentially expressed in three models (*Pla2g7*), two genes in two models (*C1qc* and *Gnb1*), and one additional gene (*C1qα*) was expressed in one of our models (*rd2*) and the bright-light-damage model [36]. These four genes are further discussed immediately below.

*PLA2G7* **(**phospholipase A2, group VII), the top-ranked gene, is localized within the BCMAD locus, a dominant form of macular dystrophy. *Pla2g7*, which is expressed specifically in mouse photoreceptors, was downregulated in all three mouse models of RD. One activity of the enzyme PLA2G7 is to hydrolyze oxidized phospholipids, which are known to be generated in photoreceptors during normal light exposure. Deficiency of plasma PLA2G7 has been shown to increase the risk of vascular disease due to its antiinflammatory properties, and its ability to control levels of oxidative stress and lipid peroxidation [7]. Variants in *PLA2G7* have also be found to be associated with the risk of asthma [37]. Three nonsynonymous polymorphisms appear to be associated with disease, the R92H, A379V, and I198T variants [38]. All three have decreased substrate affinity of PAF, which could prolong the half-life of this highly inflammatory protein [37]. Herein, we found that tissue and plasma levels of Pla2g7 might be differentially regulated; retinal degeneration was only associated with tissue, but not plasma levels of this enzyme. In a parallel study, we have confirmed that plasma levels of PLA2G7 appear not to be associated with a higher risk of AMD, as assessed in a population of the Rotterdam study [39].

*GNB1* (guanine nucleotide binding protein, beta 1), the beta-subunit of rod-specific transducin, is localized to the LCA9 and RP32 loci. RP32, a locus for autosomal recessive retinitis pigmentosa, is located between 1p13.3 and 1p21.2, and marks a severe version of RP [40]. The LCA9 locus involved in autosomal recessive Leber congenital amaurosis, has been mapped to 1p36 by linkage mapping [41]. Gao and colleagues have recently reported an association of *GNB1* intronic variants with autosomal recessive RP, as well as autosomal recessive cone-rod dystrophy [42]. On the other hand, Kitamura and colleagues have identified the *Gnb1* gene as the site of mutation responsible for autosomal dominant Rd4, and have demonstrated that haploinsufficiency is the cause of disease [43]. This would tend to rule out *GNB1* as the gene responsible for autosomal recessive LAC9 and RP32.

Complement component 1, q subcomponent, alpha and c polypeptides (*C1qα* and *C1qc*), which are upregulated in retinal degeneration, are also localized to the LCA9 and RP32 loci. C1qα and C1qc are part of the complement component C1q, which is an element of the classical complement pathway of innate immunity. The complement pathway is one of the major means by which the body recognizes foreign antigens and pathogens as well as tissue injury, ischemia, apoptosis, and necrosis (reviewed in [44]). However, in addition to important roles in normal host responses to self and foreign antigens, the complement system is increasingly recognized to be causally involved in tissue injury during ischemic, inflammatory and autoimmune diseases (reviewed in [45]). Recent genetic evidence has identified variations in the complement inhibitory protein factor H (also known as CFH) [14,16-18], as well as variations in the genes for complement factor B, C2, and C3 [17,46], as major risk factors for the disease. However, it is unclear how misregulation of the complement system leads to the observed pathology. In mouse models of retinal disease, eliminating *C1qα* neither alters the course of photoreceptor degeneration in the *rd1* mouse [47], nor changes the development of choroidal neovascularization triggered by laser photocoagulation of Bruch’s membrane [48].

### Conclusion

We have shown that the comparative genomics approach verified existing RD genes as well as identified novel RD candidate genes. This approach may be useful for focusing the search for novel genes in both RD and other diseases for which there are appropriate mouse animal models. Further studies are now needed to provide more evidence of the functionality, role, and relevance of these genes. Those studies should include sequencing of the human genes in patients with the appropriate diagnosis as well as the generation of appropriate knockout mouse strains, or elimination/activation of the targeted gene or pathway by pharmacological or molecular means. We hope to test these and other hypotheses that were generated in an unbiased and rational strategy that we systematically developed in this report.

## **Appendix 1:** Experimental data on which the selection of genes listed in Table 1 of the manuscript is based.

**A**: Gene expression data for genes that are differentially regulated in the three mouse models of retinal dystrophy—*rd1* mouse, *rd2* mouse, and light-damage (LD) in the albino mouse—as identified by DChip analysis. Gene expression data for the experimental and control group at the two experimental time points are listed as follows: columns 2–5 *rd1* mouse at postnatal days 6 and 10 (P6, P10); columns 8–11 *rd2* mouse, P14, and P21; and columns 14–17 BALB/c control and BALB/c LD at 24 h and 48 h of LD. Each value represents the average of two replicates. **B:** Differences in gene expression levels (fold change) and respective difference of the mean (Δ mean) between experimental retinas and their age-matched controls. *Rd1* retinas were analyzed from postnatal day (P) P6, P10], *rd2* retinas from P14, P21, and light-damaged retinas (LD) after 24 and 48 h of light exposure. Gene expression analysis contains procedures for strong control of false discovery rate (FDR). As indicated in the legend to Table 1, the Affymetrix Gene ID and the gene symbol represents the common denominator for table identification in Table 1 and Table 2 as well as Appendix 1, and Appendix 2.

## **Appendix 2:** Gene ontology analysis for differentially regulated genes found in unsolved locations.

Gene ontology terms that are associated with the differentially regulated genes found in unsolved locations (tabulated in Table 1-i.e., experimental list) were analyzed. Over-represented terms for the biologic processes describing these identified genes were determined by comparing them with the reference genes (i.e., all the genes present on the entire array minus the experimental list). The top GO identifications (GO ID; column 1) and GO terms (column 2) are listed to characterize as many genes possible with significant GO terms.  The genes represented by those GO terms (column 3), as well as the corresponding p-values (column 4) are listed.
